# Comprehensive Analysis Reveals Prognostic and Therapeutic Immunity-Related Biomarkers for Pediatric Metastatic Osteosarcoma

**DOI:** 10.3390/medicina60010095

**Published:** 2024-01-04

**Authors:** Jin Yuan, Shengji Yu

**Affiliations:** Department of Orthopedics, National Cancer Center, National Clinical Research Center for Cancer, Cancer Hospital, Chinese Academy of Medical Sciences, Peking Union Medical College, No. 17 Nanli, Panjiayuan, Chaoyang District, Beijing 100021, China; orthopediconcology@outlook.com

**Keywords:** pediatric metastatic osteosarcoma, prognostic biomarker, immunotherapy, differential gene expression

## Abstract

*Background and Objectives:* Osteosarcoma, the most prevalent malignant bone tumor in children and adolescents, presents a complex pathogenesis characterized by various genetic and epigenetic alterations. This study aims to identify key differentially expressed genes (DEGs) in pediatric osteosarcoma, with a focus on those influencing metastasis and patient survival. *Materials and Methods:* We utilized the GSE33382 dataset from the GEO database for a comprehensive bioinformatic analysis. This included a protein–protein interaction (PPI) network analysis, Cox regression, and Kaplan–Meier survival analysis to identify central DEGs associated with osteosarcoma metastasis and patient survival. *Results:* Our analysis identified 88 DEGs related to osteosarcoma metastasis. Among them, three survival-related central DEGs—C1QA, CD74, and HLA-DMA—were significantly linked to patient outcomes. Further correlation analysis established a strong relationship between these genes, tumor mutation burden (TMB), immune checkpoint gene expression, and overall survival. Notably, C1QA and CD74 exhibited higher expression in non-metastatic osteosarcoma cases, suggesting a potential role in disease progression. *Conclusions:* The identified DEGs, particularly C1QA, CD74, and HLA-DMA, may serve as critical biomarkers for pediatric osteosarcoma prognosis and potential targets for immunotherapy. These findings provide a deeper understanding of the molecular landscape of osteosarcoma and open new avenues for therapeutic intervention.

## 1. Introduction

Osteosarcoma, the most prevalent primary high-grade bone malignancy, predominantly impacts children and adolescents. It typically arises in the metaphysis of long bones. In the United States, the age-adjusted incidence rate of osteosarcoma is 4.4 per million individuals under the age of 24 years [1]. This disease shows a slight male predominance, with 1.4 times higher frequency than in females [2,3]. The predilection for osteosarcoma occurs in a sequence: the distal femur (43%), proximal tibia (23%), and humerus (10%) [4]; the disease is characterized by high malignancy, rapid progression, early distant metastasis, and a poor prognosis. Since the introduction of multi-agent chemotherapy in the 1970s, including cisplatin, doxorubicin, and methotrexate, adjuvant chemotherapy combined with surgical resection has improved the five-year survival rate of osteosarcoma from 20% to 70% over the past decades. Although the improved understanding and advances in multidisciplinary comprehensive treatment are acknowledged, the clinical outcomes of pediatric patients with osteosarcoma remain poor, especially those initially diagnosed with advanced metastatic disease. About one-fifth of patients with osteosarcoma will have clinically detectable metastases at the initial diagnosis, with a survival rate of only 20%, and metastases occur at a later stage in approximately 40% of patients [5]. Moreover, 80% of metastatic osteosarcoma affects the lungs, which is the main cause of osteosarcoma-related death [5]. In high-grade osteosarcoma, the most common primary malignant bone tumor, two major challenges are inherent or acquired drug resistance and the development of metastasis [6]. The development of drug resistance, both inherent and acquired, further complicates treatment, contributing to the aggressive nature of osteosarcoma and its tendency for early distant metastasis [7]. Thus, this unmet need motivates us to identify novel diagnostic and prognostic biomarkers for pediatric metastatic osteosarcoma and provide further instructions for clinical treatment.

To our best knowledge, the tumorigenesis, development, recurrence, metastasis, and clinical outcomes of tumors are associated not only with clinical and pathological characteristics but also with the molecular process and pathway of oncogenes. Osteosarcoma is characterized by complex karyotypes and a high level of genomic instability [8]. Common genetic alterations include mutations in tumor suppressor genes such as TP53 and RB1 and other involved alterations in pathways related to cell cycle regulation, apoptosis, and DNA repair mechanisms [9]. Extensive research has shown that many aberrant gene expression profiles are intimately connected to the hallmarks of osteosarcoma [10,11]. The comprehensive analysis of aberrant gene expression has important clinical significance for the early diagnosis, therapeutic management, recurrence risk, and prognosis prediction of osteosarcoma. Currently, increasing clinical research is proposing the integration of gene interaction information and signaling pathways to establish predictive models for the etiology, diagnosis, and prognosis of diseases and the patient’s response to treatment, providing molecular characterization and more accurate prediction data. Yuan et al. [11] identified four key genes (*KRT5*, *HIPK2*, *MAP3K5*, and *CD5*) closely related to the overall survival (OS) of pediatric osteosarcoma patients based on the Therapeutically Applicable Research To Generate Effective Treatments (TARGET) database. Rothzerg et al. [10] identified 12 survival-related genes as potential independent candidate prognostic genes in osteosarcoma through the analysis of genome-wide RNA-sequencing (RNA-Seq) data merged with clinical information from the TARGET database. Chen et al. [12] identified complement C1q (C1qA, C1qB, and C1qC) as biomarkers to predict the prognosis of osteosarcoma patients and provide new insights for immunotherapy. Recent advancements have revealed the critical role of the immune system in osteosarcoma pathogenesis, bringing forth new challenges and opportunities in treatment, particularly in the field of immunotherapy. The exploration of immune-related biomarkers has become increasingly relevant, providing insights into the tumor microenvironment and the potential for targeted therapies [13]. However, there is little clinical research on biomarkers identified to predict the prognosis of pediatric metastatic osteosarcoma and guide clinical immunotherapy.

Pediatric osteosarcoma, the most prevalent bone malignancy among children and adolescents, presents significant challenges in treatment, particularly due to its complex molecular pathology. Recent advancements in understanding the epidemiology and molecular mechanisms of this disease have revealed the critical roles of certain biomarkers, including *C1QA*, *CD74*, and *HLA-DMA*. These biomarkers not only contribute to the pathogenesis of osteosarcoma but also hold promise in enhancing current therapeutic strategies, especially in the realm of immunotherapy. Their diverse biological functions, ranging from immune response modulation to an influence on tumor progression, underscore their potential as targets in developing novel diagnostic and therapeutic approaches. In this study, we obtained pediatric osteosarcoma gene profiles (GSE33382) from the Gene Expression Omnibus (GEO) database. Differentially expressed genes (DEGs) between metastatic and non-metastatic pediatric osteosarcoma patients were identified using R software (v4.0.3). Subsequently, the central DEGs were analyzed using protein–protein interaction (PPI) network analysis. The prognostic biomarkers *C1QA*, *CD74*, and *HLA-DMA* for pediatric metastatic osteosarcoma were identified using RNA-sequencing data (level 3) associated with clinical information from the TARGET-osteosarcoma dataset. Furthermore, the Gene Expression Profiling Interactive Analysis (GEPIA, http://gepia.cancer-pku.cn/ accessed on 1 March 2022) online tool was utilized to validate three central DEGs through The Cancer Genome Atlas (TCGA) dataset. A significant correlation between three central DEGs and immune checkpoints and the tumor mutational burden (TMB) was observed. The present study delves into the intricate roles of *C1QA*, *CD74*, and *HLA-DMA*, exploring their interactions within the tumor microenvironment and their implications for the immunotherapy response, thereby providing new insights into the treatment of pediatric osteosarcoma.

## 2. Materials and Methods

### 2.1. Identification of DEGs

The GSE33382 dataset downloaded from the GEO database (https://www.ncbi.nlm.nih.gov/geo/ accessed on 1 March 2022) was used to investigate the differential expression of mRNAs between metastatic and non-metastatic pediatric (age < 18 years old) osteosarcoma patients, and the download data format was MINIML. Twenty-five metastatic and nine non-metastatic samples were selected. All samples were from pre-chemotherapy patients at the initial diagnosis. Metastatic patients were defined as those who developed metastases within 5 years from the initial diagnosis. The differential expression analysis of genes was performed using the “limma” package (version: 3.4.0.2) of R software (v4.0.3) [14]. “*p* < 0.05 and Log2 (Fold Change) > 1 or Log2 (Fold Change) < −1” were defined as the thresholds for the screening of the differential expression of mRNAs.

### 2.2. Protein–Protein Interaction Network Analysis

STRING (version 11.5, https://string-db.org/ accessed on 1 March 2022), covering 67,592,464 proteins and 20,052,394,042 interactions from 14,094 organisms, including Homo sapiens, was adopted for the analysis and establishment of PPI. A PPI network based on interactions between DEGs in metastatic or non-metastatic pediatric osteosarcoma patients was constructed. The PPI network was visualized by Cytoscape 3.9.1 [15] and analyzed using CytoHubba [16] to obtain the central DEGs according to the degree in the PPI network. The PPI network was constructed using nodes with an interactive confidence value > 0.7.

### 2.3. Survival-Related DEG Screening

RNA-sequencing data (level 3) and the corresponding clinical information of osteosarcoma used for the analysis were available in the TARGET database (https://ocg.cancer.gov/programs/target accessed on 1 March 2022), in which the method of acquisition and application complied with the guidelines and policies. A Kaplan–Meier (KM) survival analysis with the log-rank test was also used to compare the survival difference between the above two groups. For Kaplan–Meier curves, *p*-values and hazard ratios (HRs) with 95% confidence intervals were generated by log-rank tests and univariate Cox proportional hazards regression with the R package (survival and survminer). *p* < 0.05 was considered statistically significant.

### 2.4. Expression of Survival-Related DEGs in Sarcomas

GEPIA is a web-based tool that provides key interactive and customizable functions for researchers to analyze RNA-sequencing expression data based on TCGA and GTEx data [17]. We performed a KM survival analysis on the relative expression of central survival-related DEGs in sarcoma patients using the Kaplan–Meier Plotter database [18] (http://kmplot.com/analysis/ accessed on 1 March 2022), with HRs and the corresponding 95% CIs. Moreover, correlation analysis and multiple-gene comparison for the candidate central survival-related DEGs were also analyzed using the GEPIA.

### 2.5. Analysis of Associations between DEGs and Tumor Immunity

The correlation between 3 DEGs and the significant immune checkpoints was analyzed through Spearman correlation analysis. Given that TMB affects tumor sensitivity to immunotherapy, Spearman correlation analysis between DEGs and TMB in pan-cancer was evaluated based on TCGA. TMB was derived from the article [19] published by Vesteinn Thorsson et al. in 2018. The rank-sum test detects two sets of data, and a *p*-value of < 0.05 is considered statistically significant.

All of the above analysis methods and R packages were implemented using the R Foundation for Statistical Computing (2020) version 4.0.3. The box plot was implemented using the R software package ggplot2; principal component analysis graphs were drawn with the R software package “ggord”; the heat map was displayed using the R software package “pheatmap”; two-gene correlation graphs were implemented using the R software package ggstatsplot.

## 3. Results

### 3.1. Sample Characteristics and Identification of DEGs

**Figure 1:**
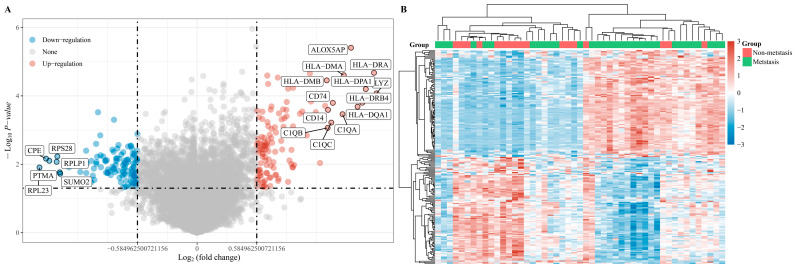
DEGs identified based on RNA-seq data from metastatic and non-metastatic pediatric osteosarcoma patients. (**A**) Volcano plots were constructed using fold-change values and *p*-values. The red points in the plot represent over-expressed mRNAs and the blue points indicate down-regulated mRNAs with statistical significance. (**B**) Heatmap of identified DEGs between metastatic and non-metastatic pediatric osteosarcoma patients.

This study examined selected samples from GSE33382, including 25 metastatic pediatric osteosarcoma samples and 9 non-metastatic samples merged with vital clinical data (Table 1). Differentially expressed genes between metastatic and non-metastatic osteosarcoma patients (fold change = 2) were compared using the “limma” package (version: 3.4.0.2) in R software. There were 52 up-regulated DEGs and 43 down-regulated DEGs (*p* < 0.05 and Log2 (fold change) > 1 or Log2 (fold change) < −1) between the patients in the two groups, and 13,933 genes were not significant (Figure 1).

### 3.2. Identification of Central DEGs by Constructing a PPI Network

**Figure 2:**
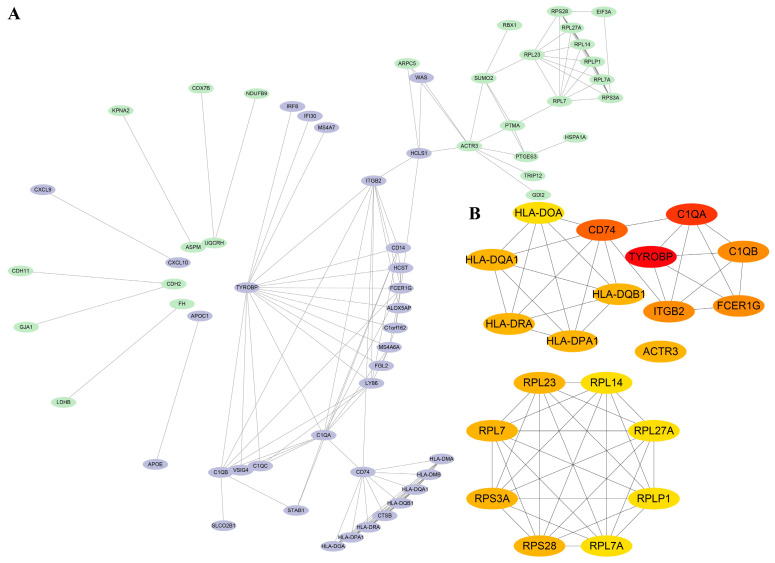
(**A**) The PPI network for 95 DEGs, in which purple is an up-regulated gene, and green is a down-regulated gene. (**B**) Twenty central DEGs in the PPI network. The red represents the degree of connectivity. The deeper the red, the higher the degree of connectivity.

The STRING database and Cytoscape 3.9.1 were applied to construct a PPI network. The PPI network constructed by STRING (Figure 2A) shows the interactions between DEGs with 62 nodes and 141 edges, and a high-confidence interaction relationship is defined by an interaction score > 0.7. The top 20 DEGs were determined as the central DEGs with the most connectedness according to the degree (Figure 2B). These central DEGs include *TYROBP*, *C1QA*, *C1QB*, *FCER1G*, *CD74*, *ITGB2*, *RPL23*, *RPL7*, *RPS28*, *RPS3A*, *HLA-DRA*, *RPL7A*, *RPLP1*, *RPL27A*, *RPL14*, *HLA-DMA*, *HLA-DMB*, *HLA-DQA1*, *HLA-DPA1*, and *HLA-DQB1*.

### 3.3. Identification of Survival-Related Central DEGs Based on the TARGET Database

**Figure 3:**
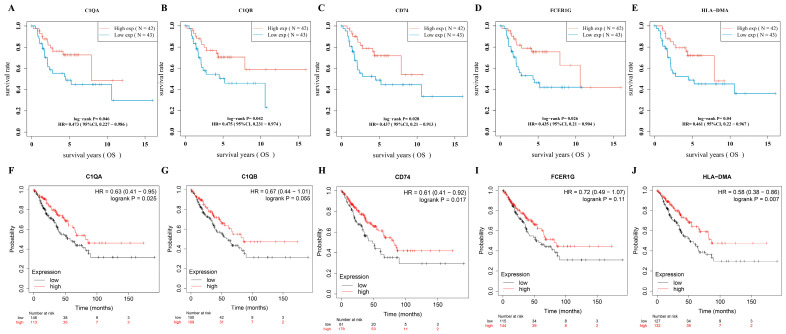
Prognostic significance of identified DEGs in pediatric osteosarcoma. (**A**) High expression of C1QA is associated with better survival (HR = 0.473, log-rank *p* = 0.046). (**B**) High expression of C1QB correlates with improved survival (HR = 0.475, log-rank *p* = 0.042). (**C**) Higher levels of CD74 expression are linked to favorable survival outcomes (HR = 0.437, log-rank *p* = 0.028). (**D**) High expression of FCER1G indicates a survival benefit (HR = 0.435, log-rank *p* = 0.026). (**E**) Elevated expression of HLA-DMA is associated with better survival (HR = 0.461, log-rank *p* = 0.04). (**F**) The high-C1QA-expression group shows a survival advantage (HR = 0.63, log-rank *p* = 0.025). (**G**) High expression of C1QB trends toward improved survival, though not statistically significant (HR = 0.67, log-rank *p* = 0.055). (**H**) The high-CD74-expression group exhibits better survival (HR = 0.61, log-rank *p* = 0.017). (**I**) The high-FCER1G-expression group tends to have better survival, but it does not reach statistical significance (HR = 0.72, log-rank *p* = 0.11). (**J**) High HLA-DMA expression significantly correlates with better survival (HR = 0.58, log-rank *p* = 0.007).

The RNA-sequencing data of osteosarcoma were downloaded from the TARGET database, and the data were merged with vital status and survival time. Twenty central DEGs were analyzed to investigate survival-related central DEGs using the TARGET osteosarcoma RNA-sequencing dataset. Log-rank tests and univariate Cox proportional hazards regression were performed using the R packages survival and survminer. Five survival-related central DEGs (*p* < 0.05; *C1QA*, *C1QB*, *FCER1G*, *CD74*, *HLA-DMA*) were found among twenty central DEGs (Figure 3A–E). To further validate survival-related central DEGs, we applied the Kaplan–Meier plotter database to analyze the association between OS and five survival-related central DEGs. We found that *C1QA* (*p* = 0.025), *CD74* (*p* = 0.017), and *HLA-DMA* (*p* = 0.007) were significantly associated with sarcoma patients’ OS (Figure 3F–J).

### 3.4. Validation and Correlation of Three Survival-Related Central DEGs

**Figure 4:**
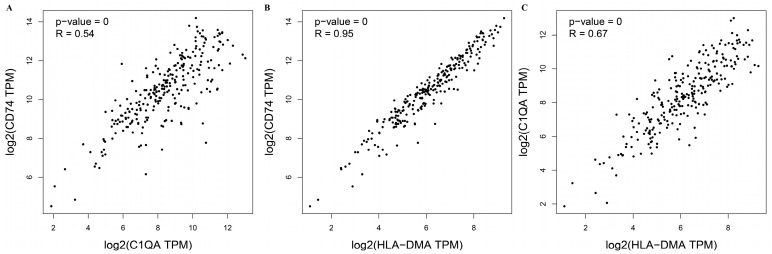
(**A**–**C**) Correlation analysis of the expression of 3 survival-related central DEGs in sarcomas.

**Figure 5:**
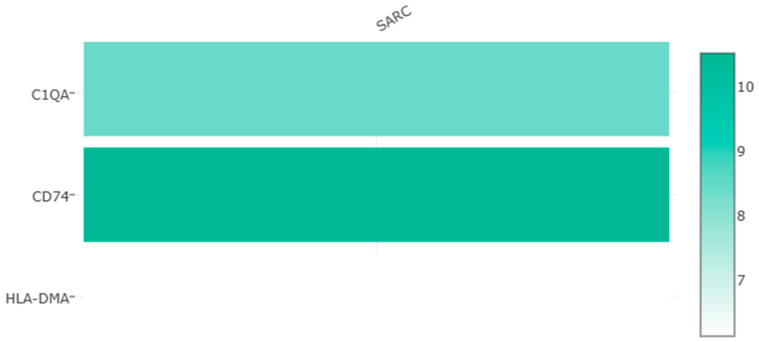
Expression levels of survival-related central DEGs in sarcomas based on GEPIA database analysis.

A correlation analysis of three survival-related central DEGs’ expression levels in sarcomas was performed based on the GEPIA database (Figure 4A–C). High significant correlations were observed between *CD74* and *HLA-DMA* (R = 0.95). Moderate positive correlations were observed between *C1QA* and *HLA-DMA* (R = 0.67). *CD74* and *C1QA* (R = 0.54) had relatively low correlations. Furthermore, a multiple-gene comparison analysis was performed using GEPIA with SARC tumor data only. *CD74* expression was the highest among the three survival-related central DEGs with a Z score of 10.5, followed by *C1QA* and *HLA-DMA* with Z scores of 8.4 and 6.1, respectively (Figure 5).

### 3.5. Correlation of DEGs and Tumor Immunity

**Figure 6:**
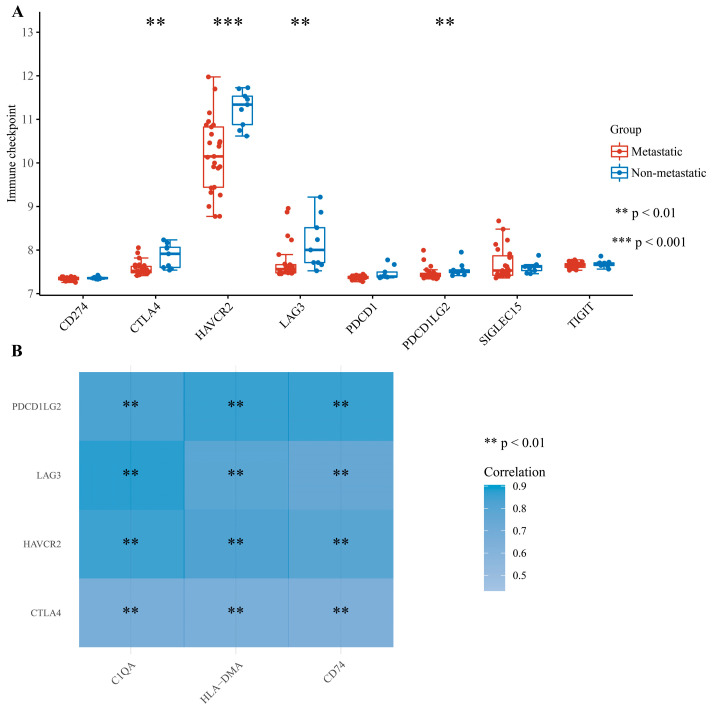
Correlation analysis of the 3 survival-related central DEGs’ expression levels with immune checkpoint gene expression. (**A**) Differential expression of immune checkpoint genes between metastatic and non-metastatic pediatric osteosarcoma patients. (**B**) Two-gene correlation or multi-gene correlation analysis between three survival-related central DEGs and four significant immune checkpoints.

**Figure 7:**
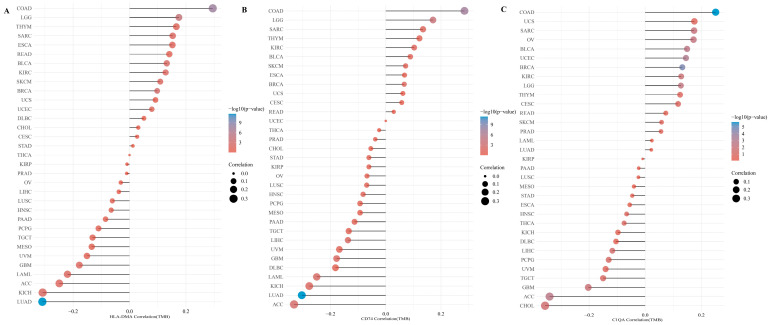
Differential correlation of TMB with DEGs in various cancers. In each subfigure, the x-axis represents the Spearman correlation coefficient with TMB, the y-axis lists the cancer types, the size of the dots indicates the strength of the correlation, and the color scale reflects the *p*-value. Darker blue dots signify stronger statistical significance of the correlation. (**A**) Correlation of HLA-DMA with TMB. Displaying a varied correlation across cancer types with a focal point on the SARC cohort where the correlation is observable, albeit not the most prominent. (**B**) Correlation of CD74 with TMB. Spearman correlation for CD74 across cancer types, with sarcomas showing a notable moderate correlation. (**C**) Correlation of C1QA with TMB. C1QA’s correlation with TMB spans across a range of cancers. The correlation within the SARC cohort is present and consistent with the overall trend seen in other cancer types.

Given that these genes are broadly involved in immune responses, we performed an extraction of the expression value of immune checkpoint genes for comparison between metastatic and non-metastatic pediatric osteosarcoma patients. The extraction and comparison of the expression values of immune checkpoint genes were conducted between metastatic and non-metastatic pediatric osteosarcoma samples from the GSE33382 dataset. Relatively low expression levels of CTLA4, HAVCR2, LAG3, and PDCD1LG2 can be observed in the group of patients with metastasis (*p* < 0.05) (Figure 6A), which may indicate and predict a poor prognosis in patients with osteosarcoma. We then evaluated the correlation between three survival-related central DEGs and four immune checkpoints, and each of the survival-related central DEGs was positively correlated with these four immune checkpoints (Figure 6B). Additionally, we investigated the correlation between three survival-related central DEGs and TMB in pan-cancer. As shown in Figure 7, the correlation of the three survival-related central DEGs and TMB in SARC data was higher than correlations for other tumor types.

## 4. Discussion

Osteosarcoma is the most common subtype of bone malignancy in children and adolescents, and the prognosis of advanced or metastatic pediatric osteosarcoma patients is poor. Unfortunately, to date, no well-established prognostic biomarkers for pediatric metastatic osteosarcoma have been identified. Moreover, osteosarcoma patients easily develop chemoresistance and tumor immune escape, promoted by cancer stem cells and the tumor microenvironment, resulting in the frequent failure to respond to conventional chemotherapy [20]. As novel treatment options, immunotherapies, including immune checkpoint inhibitors, can effectively circumvent chemoresistance in osteosarcoma. In this study, we analyzed differential gene expression associated with the metastasis of pediatric osteosarcoma within 5 years based on the GSE33382 dataset downloaded from the GEO database and identified 88 differentially expressed genes. The PPI network was visualized and analyzed to select the most central DEGs. A Cox regression analysis and KM survival analysis were performed to screen survival-related central DEGs based on the osteosarcoma project of the TARGET database, and three survival-related central DEGs were identified (*C1QA*, *CD74*, *HLA-DMA*). We then evaluated and validated the ability of these genes to serve as diagnostic and prognostic biomarkers for pediatric metastatic osteosarcoma based on multiple databases. Finally, the correlation analysis of the three survival-related central DEGs and tumor immunity revealed that these genes may guide clinical immunotherapy.

C1qA and C1qB are two of the three polypeptide chains of the complement protein C1q, which is a subcomponent of the complement classical pathway, functioning as a vital mediator in innate immunity and the adaptive immune system to combat invading pathogens [21]. Recently, the role of C1q in cancer has gradually been explored in gliomas, pancreatic cancer, and melanoma [22,23,24,25]. A previous study showed that C1q acted as a cancer-promoting factor independent of complement activation in a syngeneic murine model of melanoma, affecting angiogenesis, tumor progression, and metastasis [26]. Kemp et al. [22] reported that the up-regulation of *C1QA* and *C1QB* was observed in pancreatic ductal adenocarcinoma tumor-associated macrophages (TAMs) systemically, but the exact roles remain to be elucidated. C1q-directed macrophage polarization and M2 macrophages were associated with angiogenesis [27]. Furthermore, it was previously reported that TAMs were associated with reduced metastasis in high-grade osteosarcoma, and *C1QA* expression was higher in patients without metastases, which is consistent with our results [28]. In addition, recently, Mezheyeuski et al. expanded the understanding of C1q’s role in cancer, highlighting its association with macrophage populations and complement complex subunits in various solid tumors, thereby underscoring the potential of C1q as a marker for immune cell activity and its influence on tumor progression [29]. Thus, *C1QA* and *C1QB* may be implicated in the pathogenesis and metastasis of osteosarcoma, and the underlying mechanisms may rely on regulating macrophage polarization in osteosarcoma. Additionally, our results showed that *C1QA* was highly correlated with pediatric osteosarcoma patients’ survival, immune checkpoint gene expression, and TMB. All of these findings indicate that C1qA is a candidate metastatic and prognostic biomarker and may guide clinical immunotherapy.

CD74, a polypeptide also known as the invariant chain, was initially identified as a human lymphocyte antigen (HLA) class II chaperone that functions in antigen presentation. Moreover, the role of *CD74* has been explored in cancer, including in the regulation of migration, invasion, and cellular signaling [30,31]. Zeiner et al. demonstrated that *CD74* has an important role in the regulation of the complexity of the tumor cell HLA class II peptidome in brain metastasis and may positively predict the prognosis of the patient [30]. CD74 is the cell membrane receptor of macrophage migration inhibitory factor (MIF) [32]. A previous study showed that enhanced CD74/MIF interactions activated the PI3K/AKT pathway in melanoma and resulted in the promotion of tumor survival [33]. In addition, CD74’s role in pediatric osteosarcoma, similar to melanoma, involves critical immunological interactions. Targeting the MIF-CD74 axis, known for affecting tumor survival and immune responses, offers potential for immunotherapy, especially considering CD74’s link to a better prognosis in non-metastatic osteosarcoma [34]. In our study, we identified *CD74* as a survival-related biomarker for the prediction of a positive prognosis, with a relatively high expression in non-metastatic pediatric osteosarcoma patients. The exact reason remains to be further explored.

The *HLA-DMA* gene encodes HLA-DM-α, which is involved in antigenic peptide loading on HLA class II molecules. There are relatively few studies on the role of HLA-DMA in cancer. Previously, several studies identified HLA-DM-α as a prognostic biomarker in gastric cancer and glioblastoma [35,36]. Recently, it was demonstrated that HLA-DMA, identified as a crucial TME-related gene in lung adenocarcinoma (LUAD), correlates positively with survival and may serve as a potential biomarker for immune infiltration and response to immunotherapy [37]. Echoing our findings, recent research underscores the synergistic role of HLA-DMA in tumor immune dynamics, particularly in ER-negative breast cancer, where its expression, in concert with other immune-related genes, notably influences T-cell activity and immunotherapy outcomes, reinforcing HLA-DMA’s potential as a multifaceted biomarker in cancer immunology [38]. In the current study, the correlation analysis showed that *HLA-DMA* was closely correlated with *CD74*, with an R of 0.95. Though HLA-DM-α was identified as a prognostic biomarker in pediatric osteosarcoma in the current study, an investigation of the underlying mechanism, which may be particularly associated with CD74, is further required.

In future studies, it would be insightful to explore the interplay between the identified biomarkers and other elements of the tumor microenvironment, particularly focusing on their influence on immune checkpoint pathways and treatment resistance mechanisms. Delving deeper into the molecular interactions and signaling pathways involving C1QA, CD74, and HLA-DMA could reveal novel therapeutic targets. Additionally, investigating the applicability of these biomarkers in personalized medicine, especially in predicting the response to immunotherapy, could significantly advance treatment strategies for pediatric osteosarcoma. This exploration might offer groundbreaking insights into the disease’s pathology and pave the way for innovative, tailored treatment approaches. This research highlights the need for clinical trials to validate these biomarkers’ utility in guiding immunotherapy and potentially improving patient outcomes in pediatric osteosarcoma.

In conclusion, our research identified three novel metastasis-related DEGs associated with the survival of pediatric osteosarcoma patients: *C1QA*, *CD74*, and *HLA-DMA*. All of these genes can predict the metastasis and survival outcomes of pediatric osteosarcoma patients. In addition, these three DEGs may be closely associated with some common cellular mechanisms and immune infiltrates in osteosarcoma, especially TAMs. Further analysis of the correlation between DEGs and tumor immunity revealed that survival-related central DEGs may guide the clinical treatment application of immune checkpoint inhibitors.

## Figures and Tables

**Table 1:** Clinical characteristics of samples from pediatric osteosarcoma patients from GSE33382.

Accession	Gender	Age (Months)	Metastasis within 5 Years	Location	Histological Subtype
GSM825626	F	192	no	tibia/fibula	osteoblastic
GSM825627	F	111	no	femur	pleomorphic
GSM825631	M	200	yes	tibia/fibula	osteoblastic
GSM825633	M	177	yes	femur	osteoblastic
GSM825634	F	212	yes	femur	osteoblastic
GSM825635	M	216	no	femur	osteoblastic
GSM825636	M	200	yes	femur	telangiectatic
GSM825637	F	164	yes	tibia/fibula	telangiectatic
GSM825639	M	175	no	tibia/fibula	osteoblastic
GSM825640	M	200	yes	humerus	osteoblastic
GSM825641	M	101	no	femur	osteoblastic
GSM825644	M	136	no	tibia/fibula	osteoblastic
GSM825646	M	198	yes	tibia/fibula	osteoblastic
GSM825647	F	137	no	tibia/fibula	osteoblastic
GSM825654	M	165	yes	femur	osteoblastic
GSM825658	M	96	yes	femur	osteoblastic
GSM825659	F	128	yes	femur	osteoblastic
GSM825661	F	81	yes	humerus	osteoblastic
GSM825662	M	144	yes	tibia/fibula	chondroblastic
GSM825663	F	204	yes	femur	osteoblastic
GSM825664	M	181	yes	tibia/fibula	sclerosing
GSM825665	M	200	yes	femur	osteoblastic
GSM825666	M	205	yes	femur	chondroblastic
GSM825667	M	183	yes	femur	osteoblastic
GSM825672	F	144	yes	femur	osteoblastic
GSM825673	F	204	no	humerus	fibroblastic
GSM825676	M	174	yes	femur	giant cell-rich
GSM825677	M	162	yes	humerus	osteoblastic
GSM825678	M	170	yes	femur	osteoblastic
GSM825679	F	133	yes	femur	fibroblastic
GSM825687	M	180	yes	tibia/fibula	chondroblastic
GSM825692	F	129	yes	femur	chondroblastic
GSM825704	M	192	no	femur	osteoblastic
GSM825706	F	180	yes	femur	sclerosing

F, female; M, male. Platform: GPL10295. All samples were pre-chemotherapy high-grade osteosarcoma obtained by biopsy.

## Data Availability

The datasets analyzed for this study are available in publicly accessible repositories. The GSE33382 dataset was obtained from the GEO database. The TARGET-osteosarcoma dataset and GEPIA database were also utilized. These datasets were used under the open access guidelines provided by each database.
